# Evaluation of a Novel Point-of-Care Blood Myxovirus Resistance Protein A Measurement for the Detection of Viral Infection at the Pediatric Emergency Department

**DOI:** 10.1093/infdis/jiae367

**Published:** 2024-07-23

**Authors:** Ruut Piri, Lauri Ivaska, Anna-Maija Kujari, Ilkka Julkunen, Ville Peltola, Matti Waris

**Affiliations:** Department of Paediatrics and Adolescent Medicine, Turku University Hospital and University of Turku; Department of Paediatrics and Adolescent Medicine, Turku University Hospital and University of Turku; InFLAMES Research Flagship Center, University of Turku; Department of Paediatrics and Adolescent Medicine, Turku University Hospital and University of Turku; InFLAMES Research Flagship Center, University of Turku; Institute of Biomedicine, University of Turku and Department of Clinical Microbiology, Turku University Hospital, Turku, Finland; Department of Paediatrics and Adolescent Medicine, Turku University Hospital and University of Turku; InFLAMES Research Flagship Center, University of Turku; Institute of Biomedicine, University of Turku and Department of Clinical Microbiology, Turku University Hospital, Turku, Finland

**Keywords:** children, myxovirus resistance protein A, point-of-care test, respiratory tract infection, viral infection

## Abstract

**Background:**

Prompt differentiation of viral from bacterial infections in febrile children is pivotal in reducing antibiotic overuse. Myxovirus resistance protein A (MxA) is a promising viral biomarker.

**Methods:**

We evaluated the accuracy of a point-of-care (POC) measurement for blood MxA level compared to the reference enzyme immunoassay in 228 febrile children aged between 4 weeks and 16 years, enrolled primarily at the emergency department (ED). Furthermore, we analyzed the ability of MxA to differentiate viral from bacterial infections.

**Results:**

The mean difference between POC and reference MxA level was −76 µg/L (95% limits of agreement from −409 to 257 µg/L). Using a cutoff of 200 µg/L, POC results were uniform with the reference assay in 199 (87.3%) children. In ED-collected samples, the median POC MxA level was 571 (interquartile range [IQR], 240–955) µg/L in children with viral infections, 555 (IQR, 103–889) µg/L in children with viral-bacterial coinfections, and 25 (IQR, 25–54) µg/L in children with bacterial infections (*P* < .001). MxA cutoff of 101 µg/L differentiated between viral and bacterial infections with 92% sensitivity and 91% specificity.

**Conclusions:**

POC MxA measurement demonstrated acceptable analytical accuracy compared to the reference method, and good diagnostic accuracy as a biomarker for viral infections.

## Teaser

Quantitative point-of-care blood myxovirus resistance protein A measurement had an acceptable analytical accuracy compared to the enzyme immunoassay analysis. It differentiated viral from bacterial infections in febrile children with 92% sensitivity and 91% specificity.

In the evaluation of febrile children, biomarkers such as plasma C-reactive protein (CRP) and procalcitonin can be used to estimate the likelihood of bacterial infection but with insufficient specificity [1–8]. As viruses are often detected in asymptomatic children, multiplex polymerase chain reaction (PCR) panels cannot reliably distinguish between incidental and pathogenic virus findings [9–11]. Methods to promptly confirm a viral infection as the cause of febrile illness in children at the emergency department (ED) are lacking.

Human myxovirus resistance protein A (MxA) is an intracellular protein and a promising biomarker that is induced exclusively by type I and III interferons as a response to a wide range of viral infections [12, 13]. Blood MxA level is rapidly elevated in acute symptomatic viral infections but remains low in bacterial infections and in asymptomatic children testing positive for a respiratory virus [14–17]. We have previously reported the performance of MxA as a biomarker for symptomatic viral infection in children hospitalized with suspected severe infections [18]. Given the nonnegligible risk of viral-bacterial coinfection, it seems reasonable to use MxA in combination with a biomarker for bacterial infection.

Measuring MxA level in whole blood samples with enzyme immunoassay (EIA) takes several hours in the laboratory. However, to make rapid decisions on patient management in acute care settings, a rapid and accurate point-of-care (POC) test is critically needed. The Labmaster LUCIA Analyzer (Labmaster Ltd, Kaarina, Finland) is a POC instrument for in vitro diagnostics, including the quantitative measurement of blood MxA level within 11 minutes.

The aims of this study were (1) to compare the accuracy of a quantitative, rapid POC MxA measurement with the EIA laboratory method in the pediatric acute care setting, and (2) to assess the performance of the POC MxA measurement in distinguishing viral from bacterial infections in febrile children at the ED.

## METHODS

### Study Design and Conduct

This prospective study was conducted at the pediatric ED and the pediatric ward for infectious diseases in Turku University Hospital, Finland, from May 2020 to September 2022. Most patients present to the ED directly from the community. The study inclusion criteria were (1) age between 4 weeks and 16 years, (2) fever defined as temperature ≥38.0°C measured at the ED or within 72 hours earlier as reported by parents, and (3) capillary or venous blood sample drawn by the decision of the attending physician.

The study protocol was approved by the Ethics Committee of the Hospital District of Southwest Finland. The parents of all children, and older children or adolescents themselves, provided their written informed consent at the enrollment.

### MxA Measurements and Diagnostic Measures

Samples for POC and reference EIA test measurement for blood MxA level were collected simultaneously either by capillary or venous puncture. The POC MxA test with Labmaster LUCIA Analyzer is a sandwich immunoassay with Tb(III) chelate-labeled antibody detected by measuring cathodic electrochemiluminescence. We compared the LUCIA MxA kit to a laboratory-developed MxA EIA [17]. For the LUCIA MxA test, 7 µL of whole blood was mixed with 20 µL of lysis solution in a reaction tube and 7 µL of the lysed sample was applied to a test cassette analyzed by the instrument. The read-out is obtained after 11 minutes and has a quantitative range between 50 and 1000 µg/L. For samples collected at the ED, POC MxA test was performed by an ED physician or nurse, and for samples collected at the ward and transported to the laboratory, by a laboratory technician. Blood samples for MxA EIA reference measurement were diluted 1:20 in hypotonic buffer and stored at −70°C until the EIA was performed as described earlier [17]. CRP level was measured either from a capillary blood sample by Afinion 2 POC Analyzer (Abbott Diagnostics) or from a plasma sample by standard laboratory methods. Diagnostic and other samples (eg, blood for routine laboratory tests, nasopharyngeal sample for respiratory virus detection, or urine sample for bacterial culture) were collected, and radiographic imaging was performed if needed by the decision of the attending physician.

### Classification of Children According to Etiology

Clinical diagnoses recorded at discharge by the attending physician formed the basis for the etiologic classification. The diagnoses were verified by a review of all clinical, laboratory, and radiologic imaging data from the electronic medical records. In case of any inconsistency regarding the diagnostic decision-making, the final diagnosis was based on the expert opinion of 2 study physicians with expertise in pediatric infectious diseases who were blinded to the reference MxA EIA test results but not POC MxA results.

All study children were classified into 5 etiological groups (see Supplementary Methods for details): (1) bacterial infection: a clinical or microbiologically verified bacterial infection in the absence of viral infection; (2) viral infection: a clinical or microbiologically verified viral infection in the absence of bacterial infection; (3) viral-bacterial coinfection: an infection with suspected or microbiologically verified viral and bacterial etiology, or simultaneous viral and bacterial infections at distinct foci; (4) infection of undetermined etiology; and (5) noninfectious disease.

### Statistical Analyses

The agreement between the POC and reference EIA method results for MxA levels was analyzed according to the Bland-Altman method [19]. All POC and reference test results below the POC analyzer's measuring range (<50 µg/L) were referred to as 25 µg/L and above the upper detection limit (>1000 µg/L) as 1001 µg/L in the statistical comparison. A 2-way mixed model intraclass correlation coefficient was determined. Based on previous studies [15–18], we selected a cutoff of 200 µg/L to indicate an evident antiviral response as a marker of viral infection, and examined the proportion of children correctly classified according to this cutoff with the POC analyzer compared to the reference method. Three separate sensitivity analyses were performed: first, by excluding values exceeding the POC analyzer's measuring range (>1000 µg/L); second, by excluding children enrolled at the pediatric ward whose POC samples were analyzed by laboratory technicians; and third, by restricting analysis to children with a microbiologically confirmed etiology. In the third analysis, for children with a microbiologically confirmed viral-bacterial coinfection, microbiological evidence of both a virus and a bacterium was required.

In children recruited at the ED, POC blood MxA levels among the above-defined etiologic groups were compared using Kruskal-Wallis test followed by pairwise Mann-Whitney *U* test. *P* values were adjusted for multiple comparisons with the use of Bonferroni correction. Receiver operating characteristic (ROC) analysis was used to evaluate the capability of blood MxA level and blood MxA to CRP ratio to differentiate between viral and bacterial infections. Cutoff levels were calculated from the ROC analyses using Youden index (sensitivity + specificity – 1). We also present an alternative cutoff selected by potential clinical applicability. Two-tailed *P* values of <.05 were considered statistically significant. Statistical analyses were performed using SPSS version 27.0 software (IBM).

## RESULTS

### Study Population

We recruited 198 children from the ED and 50 children from the pediatric ward. Twenty children were excluded due to a missing sample or result, or delay of >72 hours between sampling and reference test processing, resulting in a total number of 228 children in the analyses. Of the POC samples, 188 (82.5%) were collected and analyzed by the attending ED personnel, and 40 (17.5%) were collected at the ward and analyzed by the laboratory personnel. The median age of study children was 1.9 (interquartile range [IQR], 0.8–5.4) years (Table 1). Most children (n = 142 [62.3%]) were managed as outpatients. Viral respiratory infection with or without otitis media (n = 79 [34.6%]), undetermined viral infection (n = 29 [12.7%]), and pneumonia (n = 21 [9.2%]) were the most common clinical diagnoses. Any viral sample was collected from 148 (64.9%) children for the detection of respiratory, gastrointestinal, or herpes simplex viruses. Severe acute respiratory syndrome coronavirus 2 was the most frequent viral finding, detected in 15 children.

**Table 1.** Clinical Characteristics, Diagnoses, and Detected Pathogens in 228 Study Children

Characteristic	No. (%)
Age, y, median (IQR)	1.9 (0.8–5.4)
Sex	
Male	120 (52.6)
Female	108 (47.4)
Chronic conditions	
None	173 (75.9)
Immunosuppressive disease or medication^a^	10 (4.4)
Other condition^b^	45 (19.7)
Disease characteristics	
Antibiotic treatment upon discharge	85 (37.3)
Admitted to hospital	86 (37.7)
Admitted to intensive care unit	12 (5.3)
Clinical diagnoses	
Viral respiratory infection^c^	79 (34.6)
Undetermined viral infection	29 (12.7)
Pneumonia	21 (9.2)
Pyelonephritis	18 (7.9)
Suspected or microbiologically verified sepsis without focus	12 (5.3)
Tonsillitis	12 (5.3)
Gastroenteritis	11 (4.8)
Skin or soft tissue infection	10 (4.4)
Osteomyelitis	5 (2.2)
Enteroviral disease, EBV infection, or exanthema subitum	5 (2.2)
Central nervous system infection	3 (1.1)
Appendicitis	2 (0.9)
Noninfectious disease or fever of unknown origin^d^	21 (9.2)
Any viral sample^e^ collected	148 (64.9)
Multiplex PCR respiratory sample collected	45 (19.7)
Respiratory viruses, No.	
SARS-CoV-2	15
Rhinovirus	11
Human bocavirus	5
Respiratory syncytial virus A or B	4
Parainfluenza virus 1, 2, 3, or 4	4
Human metapneumovirus	3
Adenovirus	3
Influenza virus A or B	2
Coronavirus 229E, NL63, OC43, or HKU1	1
Enterovirus	1
Other viruses, No.	
Herpesviruses^f^	4
Astrovirus	1
Bacterial species isolated from blood or other sterile site^g^, No.	
*Escherichia coli*	15
*Streptococcus* spp (*S pneumoniae*, *pyogenes*, *agalactiae*, or *anginosus*)	4
*Pseudomonas aeruginosa*	3
*Staphylococcus aureus*	3
*Enterococcus faecalis*	1
*Aerococcus urinae*	1
*Enterobacter cloacae* complex	1
*Klebsiella pneumoniae* complex	1
*Veillonella parvula*	1
*Haemophilus parainfluenzae*	1

Data are presented as No. (%) unless otherwise indicated.

Abbreviations: EBV, Epstein-Barr virus; IQR, interquartile range; PCR, polymerase chain reaction; SARS-CoV-2, severe acute respiratory syndrome coronavirus 2.

^a^Hematologic disorder (n = 4), rheumatologic disorder (n = 2), gastrointestinal or hepatic disorder (n = 2), primary cilia dyskinesia (n = 1), heart transplant (n = 1).

^b^Asthma (n = 16), neurological disorder or syndrome (n = 7), cardiovascular disease (n = 5), endocrine disorder (n = 4), urological or renal disorder (n = 4), birth at <32 weeks (n = 4), gastrointestinal disorder (n = 2), hematologic disorder (n = 1), rheumatologic condition (n = 1), or other (n = 1).

^c^Upper respiratory tract infection, bronchiolitis, wheezy bronchitis, laryngitis, SARS-CoV-2 infection, or influenza with or without otitis media or other localized bacterial complication.

^d^Fever of unknown origin (n = 12), erythema multiforme/allergic reaction (n = 3), multisystem inflammatory syndrome in children (n = 1), inflammatory bowel disease (n = 1), nonspecific diarrhea of infancy (n = 2), periodic fever (n = 1).

^e^Respiratory multiplex PCR or antigen detection assay, SARS-CoV-2 PCR assay, combined SARS-CoV-2, respiratory syncytial virus and influenza PCR assay, herpes simplex virus PCR assay, or stool multiplex PCR assay.

^f^Herpes simplex virus or EBV.

^g^Urine, cerebrospinal fluid, pleural fluid, or abscess fluid.

### Analytical Accuracy of POC MxA Measurement

**Figure 1.**
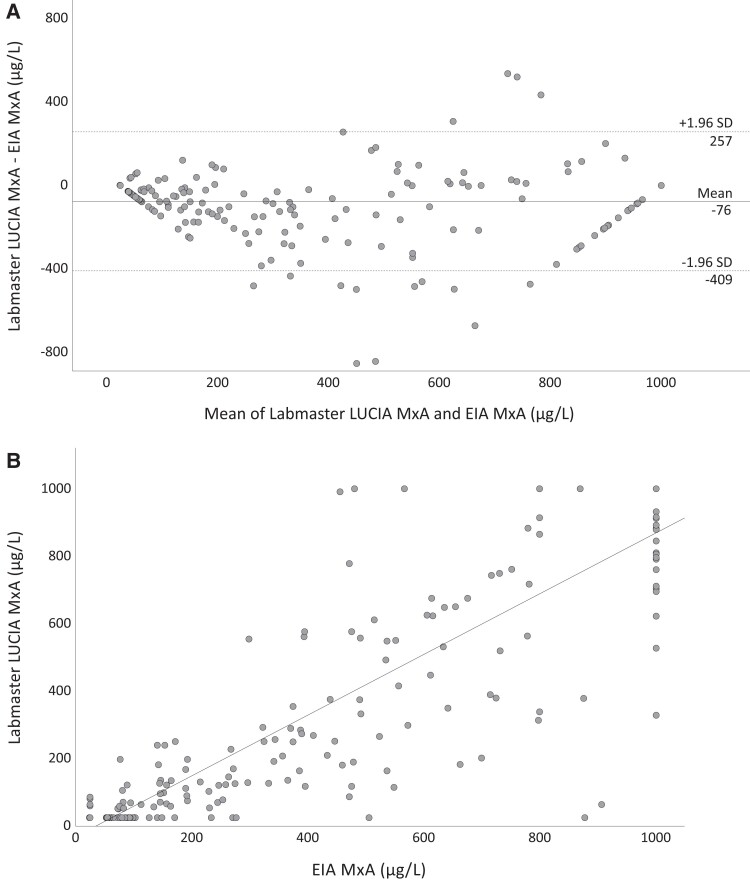
Bland-Altman and linear correlation plots for comparison of blood myxovirus resistance protein A (MxA) levels measured by point-of-care (POC) and reference methods in 228 febrile children. *A*, Bland-Altman plot presenting level of agreement between the POC Labmaster LUCIA MxA measurement and the reference enzyme immunoassay (EIA) analysis. The mean difference (−76 µg/L) between the methods is represented as a solid line and the 95% limits of agreement (from −409 to 257 µg/L) as dashed lines. *B*, Scatterplot showing linear correlation between the POC Labmaster LUCIA MxA measurement and the reference EIA analysis. Of 228 samples of febrile children, 188 were collected and analyzed by the attending pediatric emergency department personnel, and 40 were collected at the pediatric ward for infectious diseases and analyzed by the laboratory personnel. Abbreviations: EIA, enzyme immunoassay; MxA, myxovirus resistance protein A; SD, standard deviation.

The mean difference between the POC and reference MxA levels was −76 µg/L with 95% limits of agreement from −409 to 257 µg/L (Figure 1*A*). Figure 1*B* represents the respective linear correlation. Single measure intraclass correlation between the POC and reference method was 0.878 (95% confidence interval [CI], .799–.921). Using a cutoff of 200 µg/L as an indicator of an antiviral response, POC MxA results were uniform with the reference assay in 199 (87.3%) children. Of 111 POC MxA values above the cutoff, 109 (98.2%) were consistent with the reference method. Clinical features of 29 children with discordant results between the POC and EIA MxA measurements are shown in Supplementary Table 1.

When POC values (n = 35) exceeding the upper limit of the LUCIA Analyzer's measuring range (>1000 µg/L) were excluded, the mean difference between the POC and reference MxA levels was −96 µg/L with 95% limits of agreement from −429 to 237 µg/L. In children with POC MxA result >1000 µg/L, the values by the reference method varied from 481 µg/L to 4684 µg/L (median, 2264 µg/L).

In a sensitivity analysis restricted to children (n = 188) recruited at the ED, the agreement between the POC and reference test results remained similar. The mean difference between the POC and reference test MxA levels was −78 µg/L with 95% limits of agreement from −431 to 275 µg/L. With a cutoff of 200 µg/L, POC MxA results were uniform with the reference assay in 168 (89.4%) children. Characteristics of these children are shown in Supplementary Table 2.

### POC MxA Levels in the Differentiation Between Viral and Bacterial Infections

**Figure 2.**
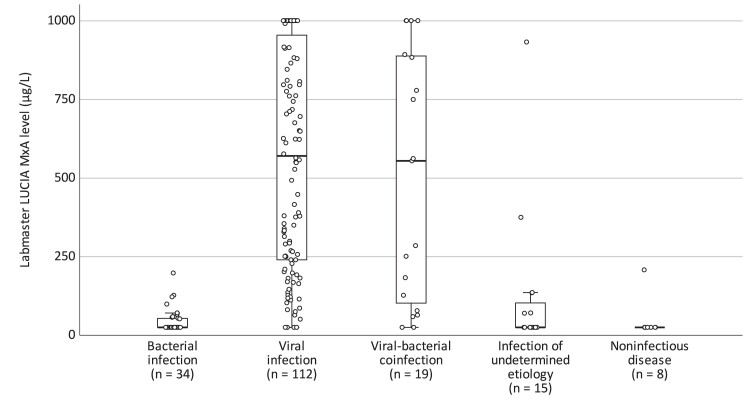
Point-of-care Labmaster LUCIA myxovirus resistance protein A (MxA) levels in 188 febrile children at the emergency department, by etiology. For each group, the horizontal line represents the median, the box the upper and lower quartiles, and the whiskers the 95% confidence interval. For pairwise comparisons of groups “Viral infection” and “Viral-bacterial coinfection” with “Bacterial infection,” *P* < .001 for both comparisons by Mann-Whitney *U* test.

We compared POC MxA levels in 188 children enrolled at the ED in different groups divided by the etiology of the infection. We determined 112 (59.6%) children to have a viral infection, 34 (18.1%) a bacterial infection, 19 (10.1%) a viral-bacterial coinfection, 15 (8.0%) an infection of undetermined etiology, and 8 (4.3%) a noninfectious disease. Characteristics of these children according to the etiologic group are shown in Supplementary Table 3. POC MxA levels (median [IQR]) were higher in children with a viral infection (571 [240–955] µg/L) compared to children with a bacterial infection (25 [25–54] µg/L, *P* < .001) or a noninfectious disease (25 [25–25] µg/L, *P* < .001) (Figure 2). In children with a viral-bacterial coinfection, the blood MxA levels were similar (median 555 [IQR, 103–889] µg/L, *P =* 1.00) to children with a viral infection only. Clinical features of 30 ED-recruited children with discordance between the POC MxA result and adjudicated etiology of infection are shown in Supplementary Table 4.

**Figure 3.**
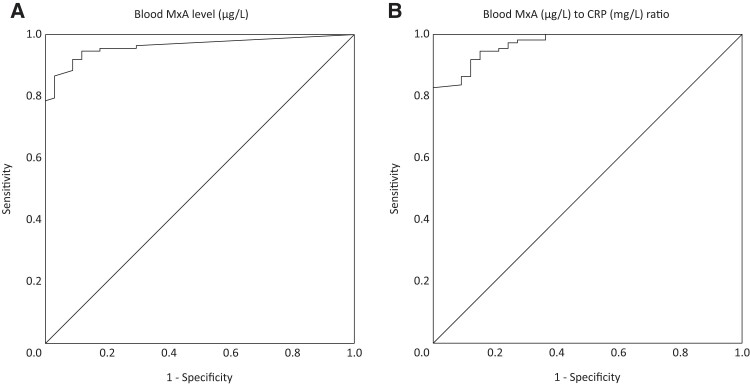
Differentiation between viral and bacterial infections by point-of-care (POC) Labmaster LUCIA myxovirus resistance protein A (MxA) and MxA to C-reactive protein (CRP) ratio in febrile children at the emergency department (ED). Receiver operating characteristic (ROC) curves for differentiating between viral (n = 112) and bacterial (n = 34 for *A* and n = 33 for *B*) infections in febrile children at the ED. *A*, ROC curve for POC MxA level. Area under the curve (AUC), 0.96 (95% confidence interval [CI], .94–.99). *B*, ROC curve for POC blood MxA (µg/L) to CRP (mg/L) ratio. AUC, 0.97 (95% CI, .95–.99).

In a ROC analysis for differentiation between viral (n = 112) and bacterial (n = 34) infections, blood MxA level determined by the POC method resulted in the area under the curve (AUC) of 0.96 (95% CI, .94–.99) (Figure 3*A*). The greatest sum of sensitivity (92.0%) and specificity (91.2%) for viral infections was obtained with a cutoff level of 101 µg/L, whereas a sensitivity of 78.6% and specificity of 100.0% were obtained with a cutoff level of 200 µg/L. The discriminative performance of MxA was enhanced when combined with CRP measurement. In a ROC analysis, the blood MxA (µg/L) to CRP (mg/L) ratio gave the AUC of 0.97 (95% CI, .95–.99) for differentiation between viral (n = 110) and bacterial (n = 33) infections (Figure 3*B*). The greatest sum of sensitivity (82.7%) and specificity (100.0%) was obtained with a cutoff level of 10.6.

### POC MxA Levels in Children With Microbiologically Confirmed Etiology

Among the 188 children enrolled at the ED, 40 (21.3%) had a microbiologically confirmed etiology of infection. POC MxA levels (median [IQR]) were higher in children (n = 18) with a microbiologically confirmed viral infection without bacterial infection (785 [558–1001] µg/L) compared to children (n = 17) with a bacterial infection without viral infection (25 [25–25] µg/L, *P* < .001) (Supplementary Figure 1). The median (IQR) MxA level of children (n = 5) with a microbiologically verified viral-bacterial coinfection was 251 (59–562) µg/L (*P* = .074 and *P* = .14 compared to the levels in confirmed bacterial or viral infections, respectively).

In a ROC analysis for differentiation between microbiologically proven viral (n = 18) and bacterial (n = 17) infections, POC MxA level and POC MxA (µg/L) to CRP (mg/L) ratio resulted in AUCs of 0.96 (95% CI, .89–1.00) and 0.99 (95% CI, .97–1.00), respectively (Supplementary Figure 2). The optimal cutoffs regarding the highest sums of sensitivity (88.9% and 94.4%, respectively) and specificity (100.0% for both) were 232 µg/L for POC MxA level and 15.2 for POC MxA (µg/L) to CRP (mg/L) ratio.

### Association of Blood MxA Levels With Age and Need for Hospitalization

We examined the effect of age on MxA responses in ED-recruited children with a viral infection with or without a simultaneous bacterial infection. The median (IQR) POC MxA level was higher (704 [299–1001] µg/L) in children <2 years of age (n = 81) compared to older children (292 [164–776] µg/L, n = 50; *P* < .001). In children with a viral infection only, MxA levels did not differ between outpatients (595 [250–992] µg/L, n = 102) compared to those (n = 10) admitted to hospital (412 [182–807] µg/L, *P* = .50).

### Practical Feasibility

For the attending physician or nurse, POC testing required approximately 2 minutes in total for sampling and sample preparation (diluting the sample in buffer solution and transferring to a test cassette) and 11 minutes for processing with the LUCIA Analyzer. Thus, POC test results were available within 15 minutes from the beginning of sampling. Technical errors in the POC analyses were rare. The feedback from the ED personnel on the use of POC test was generally positive. Negative feedback included the need for sample preparation and reading a near-field communication card before each analysis.

## DISCUSSION

In this study, we observed minor to moderate differences in quantitative blood MxA levels obtained by the rapid POC analyzer and the laboratory reference EIA method. With a cutoff limit of 200 µg/L as an indicator of an antiviral response, the POC MxA results were uniform with the reference assay in 87% of all children, and in 98% of children with POC values above the cutoff. Furthermore, we found that the MxA level measured by the POC test was specifically increased in children with an acute viral infection, supporting its clinical usability as a biomarker of viral infections.

Although some variation was noted in the POC MxA results compared to the reference method, we consider that the analytical accuracy of the POC test was adequate to confirm an antiviral response in the clinical context. We noticed that the POC values were on average slightly lower than the respective reference values. Thus, a positive value (>200 µg/L) in the POC test more strongly supports the presence of a viral infection if the same cutoff limits are used both in the POC and reference EIA tests. In a sensitivity analysis restricted to samples collected and analyzed by the ED personnel instead of laboratory technicians, the accuracy of the POC test remained similar to the main analysis. This supports the robustness of the method and its suitability to the challenging environment of a pediatric ED. Furthermore, the POC test was feasible to be used at the ED, and the test results were rapidly available.

We observed substantially increased POC MxA levels in children with a viral infection with or without a concomitant bacterial infection, whereas children with a bacterial infection only had consistently low MxA levels. The ability of MxA to discriminate between viral and bacterial infections, excluding viral-bacterial coinfections, was excellent in our study population. Previous studies, which evaluated its discriminative performance in various viral and bacterial infections, reported sensitivities between 72% and 96% and specificities between 67% and 100%, depending on the study design [15, 16, 18, 20, 21]. A recent study, which combined patients with bacterial infection and viral-bacterial coinfection in the analysis, observed good sensitivity but poor specificity in the differentiation between bacterial and viral infections [22]. Overall, the performance of MxA has been better when investigating children with clearly defined disease entities or microbiologically verified infections compared to heterogeneous populations.

Children have been reported to have higher baseline MxA values than adults, ranging from 3 to 185 µg/L [15–17, 20], and as demonstrated also in the current study, those <1–2 years of age to have stronger antiviral MxA responses compared to older children [18, 20]. In previous studies, the optimal cutoff for MxA in children has varied between 175 and 430 µg/L depending on the characteristics of the study population and whether the main objective has been on differentiating between viral and bacterial infections, or healthy and infected children [15–18, 20, 23]. Compared to our previous report in children hospitalized with a suspected severe infection [18], the best-performing MxA cutoff was lower in the present study (256 vs 101 µg/L, respectively). These differences are related to study designs. In the current study, children were mostly managed as outpatients for common childhood infections, whereas the previous study population was dominated by severe illnesses, complicated clinical presentations, and coinfections.

Since high specificity rather than high sensitivity is more important for a viral biomarker in minimizing the risk of undertreating a bacterial infection, a cutoff of 200 µg/L with a 100% diagnostic specificity and an excellent agreement between the Labmaster LUCIA MxA and reference test results seems applicable in clinical practice. However, as determining a single optimal cutoff for blood MxA level in children is challenging, quantitative numerical measurement is an advantage, since the specificity of the test improves with increasing values. Our results are novel, as this study represents the first evaluation of a quantitative POC test for MxA measurement. An international standard of the World Health Organization for MxA would greatly facilitate establishment of reference ranges and comparison between different methods and laboratories.

In line with previous findings reported by us and others [15, 18, 21, 22], the combined quantitative measurement of MxA and CRP yielded a better performance than MxA concentration alone. A combined qualitative POC measurement of MxA and CRP (FebriDx, Lumos Diagnostics, Sarasota, Florida) has mainly been studied in adults with acute respiratory infections [24–33]. FebriDx provides qualitative results based on a fairly low MxA cutoff of 40 µg/L. Notably, in a large study assessing the discriminative ability of 98 various viral and bacterial biomarker combinations, 13 best-performing combinations all included MxA, 1 of 7 individual biomarkers studied [22].

Timely access to accurate diagnostic tests is a prerequisite for the appropriate use of antibiotics. In addition to needing fewer resources and training of personnel, capillary blood sampling for POC testing is faster and less invasive compared to venous sampling. The highest added value of POC measurement for MxA is likely obtained from improving the diagnostic workup of children, particularly infants, with fever without a source. In such cases, by promptly confirming a viral infection with quantitative MxA measurement, possibly in combination with low CRP or procalcitonin level, it might be possible to reduce both additional diagnostic testing and unnecessary use of antibiotics.

A strength of our study is that we evaluated POC MxA measurement at the actual site of care, at the pediatric ED. Since we included children with a wide age range, comorbidities, and heterogeneous disease etiologies, our study population represents the real-world patient population. As a limitation of our study, the attending physicians mostly performed POC MxA tests by themselves, and hence, were not blinded to MxA results. However, as MxA measurement is not in routine use in our unit and no instructions on the interpretation of the result were given, this is not expected to have a substantial effect on the diagnostic decision-making. Also, respiratory virus multiplex PCR testing was not routinely performed on all children included in the study. Thus, etiologic determination was primarily based on clinical presentation rather than on microbiological confirmation. Despite this, the discriminatory power of MxA was excellent even though some cases that were classified as having solely a bacterial infection probably would have been virus positive if universal virus testing had been used. In a sensitivity analysis, the performance of MxA was similar or slightly improved when excluding children without a microbiologically verified etiology. Our study population included only children with a capillary or venous blood sample drawn because of other reasons, which limits the generalizability of our results, as children with mild clinical presentations and no need for blood tests were not included in the study.

In conclusion, POC measurement of blood MxA level was feasible to perform at the ED and demonstrated acceptable accuracy compared to the reference EIA method. In febrile children at the ED, POC MxA had good diagnostic accuracy as a biomarker for viral infections. Our results support the use of POC MxA measurement in identifying viral infections in pediatric acute care settings with the aim of improved antimicrobial stewardship. Further studies are needed to evaluate the ability of POC MxA measurement to truly impact diagnostic decision-making, and thus decrease unnecessary additional diagnostic testing and antibiotic use.

## Supplementary Data

Supplementary materials are available at *The Journal of Infectious Diseases* online (http://jid.oxfordjournals.org/). Supplementary materials consist of data provided by the author that are published to benefit the reader. The posted materials are not copyedited. The contents of all supplementary data are the sole responsibility of the authors. Questions or messages regarding errors should be addressed to the author.

## Supplementary Material

jiae367_Supplementary_Data

## Notes

***Author contributions.*** Concept and design: R. P., L. I., V. P., and M. W. Methodology: R. P., L. I., I. J., V. P., and M. W. Acquisition and interpretation of data: all authors. Statistical analyses: R. P. Drafting of the manuscript: R. P. and V. P. Critical revision of the manuscript for important intellectual content: all authors. Supervision: M. W. and V. P. All authors approved the final version of the manuscript to be published.

***Ethics approval.*** All procedures involving human participants were in accordance with the 1964 Helsinki declaration and its later amendments or comparable ethical standards. The Ethics Committee of the Hospital District of Southwest Finland approved the study (approval number 2/1801/2020).

***Data availability.*** The data that support the findings of this study are not openly available due to reasons of sensitivity.

***Disclaimer.*** The funding sources had no role in the design and conduct of the study; collection, management, analysis, and interpretation of the data; preparation, review, or approval of the manuscript; or decision to submit the manuscript for publication.

***Financial support*.** This work was supported by University of Turku (to R. P.); research funds from specified government transfers to Hospital District of Southwest Finland (to R. P. and V. P.); the Foundation for Pediatric Research (to R. P. and V. P.); Maud Kuistila Memorial Foundation (2021-0105B to R. P.); Päivikki and Sakari Sohlberg Foundation (230055 to R. P.); and Labmaster Ltd (to I. J. and M. W). Labmaster Ltd. provided the LUCIA Analyzers and in part the MxA kits for the study.
