# Supplementary material for: Evaluation of a Novel Point-of-Care Blood Myxovirus Resistance Protein A Measurement for the Detection of Viral Infection at the Pediatric Emergency Department
Source: J Infect Dis. 2024 Jul 23;230(5):e1049–57. doi: 10.1093/infdis/jiae367 (PMC11565905; doi:10.1093/infdis/jiae367)
Supplement: jiae367_Supplementary_Data [file jiae367_supplementary_data.docx]

**Supplementary Material**

**Evaluation of a Novel Point-of-Care Blood Myxovirus Resistance Protein A Measurement for the Detection of Viral Infection at the Pediatric Emergency Department**

Ruut Piri, Lauri Ivaska, Anna-Maija Kujari, Ilkka Julkunen, Ville Peltola, Matti Waris

**Supplementary Methods.** Definitions for Etiologic Groups1

**Supplementary Table 1.** Clinical Features of 29 Children with Discordant Results Between the Point-of-Care and Reference MxA Measurements, Based on the Cutoff of 200 µg/L 2

**Supplementary Table 2.** Clinical Characteristics, Diagnoses, and Detected Pathogens in 188 Study Children Enrolled at the Pediatric Emergency Department 5

**Supplementary Table 3.** Clinical Characteristics, Diagnoses and Detected Pathogens in 188 ED-Recruited Children According to the Etiologic Group 7

**Supplementary Table 4.** Clinical Features of 30 ED-recruited Children with Discordant Results Between the Point-of-Care MxA Result and Adjudicated Etiology of Infection, Based on the Cutoff of 200 µg/L9

**Supplementary Fig. 1.** Point-of-Care Labmaster LUCIA MxA Levels in 40 ED-Recruited Children with Microbiologically Confirmed Etiology11

**Supplementary Fig. 2.** Differentiation between Viral and Bacterial Infections by Point-of-Care Labmaster LUCIA MxA and MxA to CRP Ratio in 40 ED-Recruited Children with Microbiologically Confirmed Etiology 12

**Supplementary Methods.** Definitions for Etiologic Groups

Etiologic groups and their definitions were decided *a priori.* Clinical diagnoses recorded at discharge by the attending clinician formed the basis for the classification. The diagnoses were verified by review of all clinical, laboratory, and radiologic imaging data from the electronic medical records. If there was any inconsistency regarding the diagnostic decision making, the final diagnosis was based on the expert opinion of two study physicians with expertise in pediatric infectious diseases who were blinded to the reference MxA enzyme immunoassay (EIA) test results but not point-of-care (POC) MxA results. In cases where plasma C-reactive protein (CRP) or procalcitonin (PCT) values were utilized in the diagnostics, cutoff levels were 40 mg/L for CRP and 0.5 µg/L for PCT.

*Bacterial infection:* 1) Clinical or microbiologically verified diagnosis of sepsis, bacterial meningitis, bacterial type pneumonia (dense infiltration in x-ray and high CRP), pyelonephritis, septic arthritis, osteomyelitis, or other focal pyogenic infection, and 2) No clinical or microbiological diagnosis of viral infection.

*Viral infection:* 1) Clinical or microbiologically verified diagnosis of upper respiratory tract infection, stomatitis, pharyngitis, tonsillitis, laryngitis, bronchiolitis, wheezy bronchitis, asthma exacerbation during viral respiratory infection, influenza, chickenpox, enteroviral disease, viral meningitis, or other clinically defined viral infection, and 2) No indication of bacterial etiology in microbiologic, hematologic, chemistry, or radiologic studies.

*Viral-bacterial co-infection*: An infection with viral and bacterial etiology, or with simultaneous viral and bacterial infections at distinct foci.

*Infection of undetermined etiology*: An infectious disease that could not be categorized according to the above-mentioned criteria.

*Non-infectious disease*: A diagnosis other than an infectious disease.

**Supplementary Table 1.** Clinical Features of 29 Children with Discordant Results Between the Point-of-Care and Reference MxA Measurements, Based on the Cutoff of 200 µg/L

| POC MxA (µg/L) | Reference MxA (µg/L) | Adjudicated Etiology | Clinical Diagnosis | Age (y) | Symptoms | Detected Pathogens (Source) | Highest CRP (mg/L) | Microbiological Tests with Negative Results |
| --- | --- | --- | --- | --- | --- | --- | --- | --- |
| 183 | 663 | Co-infection | URTI, otitis media | 1 | Fever, respiratory symptoms | None | 22 | SARS-CoV2 PCR |
| < 50 | 271 | Bacterial | Pyelonephritis | 0.1 | Fever | *Escherichia coli* (urine) | 100 | SARS-CoV2 PCR, blood bacterial culture |
| < 50 | 234 | Viral | Tonsillitis/mononucleosis | 15 | Fever, sore throat | Epstein-Barr virus (serum serology) | 26 | SARS-CoV2 PCR, throat bacterial culture, serum CMV serology |
| < 50 | 878 | Viral | Undetermined viral infection, suspected roseola | 0.8 | Fever, leukopenia | None | 3 | SARS-CoV2 PCR |
| < 50 | 506 | Bacterial | Pneumonia | 1 | Fever, respiratory symptoms | None | 132 | - |
| < 50 | 277 | Bacterial | Undetermined bacterial infection | 6 | Fever | None | > 200 | Urine and blood bacterial culture |
| 146 | 264 | Viral | Undetermined viral infection | 7 | Fever | None | 79 | Urine bacterial culture, SARS-CoV2 PCR |
| 54 | 231 | Bacterial | Undetermined bacterial infection | 7 | Fever, headache, diarrhea | None | 325 | Respiratory viral and fecal bacterial multiplex PCR, throat and blood bacterial culture |
| 103 | 230 | Viral | Bronchiolitis | 0.8 | Fever, respiratory symptoms | None | 8 | SARS-CoV2, RSV and influenza PCR |
| 64 | 907 | Viral | URTI | 3 | Fever, respiratory symptoms | None | 14 | SARS-CoV2 PCR |
| 181 | 460 | Viral | URTI | 14 | Fever, respiratory symptoms, muscle pain | None | 13 | SARS-CoV2 PCR |
| 78 | 254 | Co-infection | Lobar pneumonia | 1 | Fever, respiratory symptoms | Rhinovirus, parainfluenza virus 3 (nasopharynx) | 341 | Respiratory viral multiplex PCR for other viruses, blood bacterial culture |
| 127 | 333 | Viral | Obstructive bronchitis | 1 | Fever, respiratory symptoms | None | 25 | - |
| 136 | 366 | Infection of undetermined etiology | Infection of undetermined etiology | 0.2 | Rapidly resolving low-grade fever (38.0 °C) | None | 3 | Urine bacterial culture |
| 192 | 342 | Viral | URTI | 8 | Fever, respiratory symptoms | None | 9 | - |
| 240 | 141 | Viral | Undetermined viral infection | 2 | Fever, occasional vomiting, leukopenia | None | 35 | - |
| 164 | 537 | Viral | Gastroenteritis | 7 | Fever, vomiting | None | 3 | - |
| 251 | 172 | Viral | Undetermined viral infection | 0.2 | Fever | None | 3 | Respiratory multiplex antigen detection test, blood and urine bacterial culture |
| 170 | 272 | Viral | Undetermined viral infection | 2 | Fever, headache, leukopenia | None | < 1 | Respiratory multiplex antigen detection test |
| 118 | 396 | Viral | Gastroenteritis | 0.1 | Fever, diarrhea | Adenovirus (feces) | 3 | Fecal rotavirus and norovirus PCR |
| 121 | 247 | Viral | Obstructive bronchitis | 4 | Fever, respiratory symptoms | None | - | SARS-CoV2 PCR |
| 87 | 472 | Bacterial | Undetermined bacterial infection | 0.1 | Fever | None | 78 | Respiratory and fecal viral multiplex PCR, urine and blood bacterial culture, CSF bacterial culture and PCR for entero- and herpesviruses |
| 118 | 476 | Viral | Obstructive bronchitis | 3 | Fever, respiratory symptoms | Parainfluenza virus 3 (nasopharynx) | 6 | Respiratory viral multiplex antigen detection test for other viruses |
| 164 | 386 | Viral | Laryngitis | 0.4 | Fever, respiratory symptoms | None | 30 | SARS-CoV2 PCR |
| 190 | 479 | Viral | URTI | 1 | Fever, mild respiratory symptoms | Rhinovirus (nasopharynx) | 24 | Urine and blood bacterial culture, respiratory viral multiplex PCR for other viruses |
| 126 | 275 | Viral | URTI | 10 | Fever, respiratory symptoms | SARS-CoV2 (nasopharynx) | < 1 | Blood bacterial culture |
| 129 | 297 | Bacterial | Impetigo/cellulitis | 0.9 | Fever, impetigo/cellulitis post scabies treatment | *Streptococcus pyogenes* (pus) | 90 | Blood bacterial culture |
| 131 | 215 | Bacterial | Lobar pneumonia | 7 | Fever, respiratory symptoms | None | 238 | Respiratory viral multiplex PCR, blood bacterial culture |
| 123 | 259 | Viral | Adenoviral infection | 0.2 | Fever, vomiting | Adenovirus (nasopharynx) | 41 | Respiratory viral multiplex PCR for other viruses, blood bacterial culture |

CRP – C-reactive protein

URTI – upper respiratory tract infection

PCR – polymerase chain reaction

CMV - Cytomegalovirus

US – ultrasound

MRI – magnetic resonance imaging

RSV – respiratory syncytial virus

CSF – cerebrospinal fluid

The first 20 patients recruited at the emergency department, and the last nine at the pediatric ward, with cases listed in chronological order within these groups

**Supplementary Table 2.** Clinical Characteristics, Diagnoses, and Detected Pathogens in 188 Study Children Enrolled at the Pediatric Emergency Department

| Age (yrs), median (interquartile range, IQR) | 1.7 (0.7–4.7) |
| --- | --- |
| Sex, *n* (%) |  |
| Male | 96 (51.1) |
| Female | 92 (48.9) |
| Chronic conditions, *n* (%) |  |
| None | 147 (78.2) |
| Immunosuppressive disease or medication^a^ | 7 (3.7) |
| Other condition^b^ | 34 (18.1) |
| Disease characteristics, *n* (%) |  |
| Antibiotic treatment upon discharge | 59 (31.4) |
| Admitted to hospital | 46 (24.5) |
| Admitted to intensive care unit | 8 (4.3) |
| Clinical diagnoses, *n* (%) |  |
| Viral respiratory infection^c^ | 67 (35.6) |
| Undetermined viral infection | 29 (15.4) |
| Pneumonia | 15 (8.0) |
| Pyelonephritis | 16 (8.5) |
| Suspected or microbiologically verified sepsis without focus | 7 (3.7) |
| Tonsillitis | 12 (6.4) |
| Gastroenteritis | 11 (5.9) |
| Skin or soft tissue infection | 4 (2.1) |
| Osteomyelitis | 1 (0.5) |
| Enteroviral disease, Epstein-Barr virus infection, or exanthema subitum | 4 (2.1) |
| Central nervous system infection | 3 (1.6) |
| Appendicitis | 1 (0.5) |
| Non-infectious disease or fever of unknown origin^d^ | 18 (9.5) |
| Any viral sample^e^ collected, *n* (%) | 111 (59.0) |
| Multiplex PCR respiratory sample collected, n (%) | 24 (12.8) |
| Respiratory viruses, *n* |  |
| SARS-CoV-2 | 14 |
| Rhinovirus | 4 |
| Respiratory syncytial virus A or B | 4 |
| Human bocavirus | 2 |
| Parainfluenza virus 1, 2, 3, or 4 | 2 |
| Human metapneumovirus | 2 |
| Adenovirus | 2 |
| Influenza virus A or B | 2 |
| Coronavirus 229E, NL63, OC43, or HKU1 | 1 |
| Enterovirus | 1 |
| Other viruses, *n* |  |
| Herpesviruses^f^ | 3 |
| Astrovirus | 1 |
| Bacterial species isolated from blood or other sterile site^g^, *n* |  |
| *Escherichia coli* | 12 |
| *Pseudomonas aeruginosa* | 2 |
| *Streptococcus* species (*S. pneumoniae* or *agalactiae*) | 2 |
| *Enterococcus faecalis* | 1 |
| *Aerococcus urinae* | 1 |
| *Enterobacter cloacae complex* | 1 |
| *Klebsiella pneumoniae complex* | 1 |

^a^ Hematologic disorder (*n* = 3), rheumatologic disorder (*n* = 1), gastrointestinal or hepatic disorder (*n* = 1), primary cilia dyskinesia (*n* = 1), heart transplant (*n* = 1)

^b^ Asthma (*n* = 8), neurological disorder or syndrome (*n* = 7), cardiovascular disease (*n* = 5), endocrine disorder (*n* = 1), urological or renal disorder (*n* = 4), birth at <32 weeks (*n* = 4), gastrointestinal disorder (*n* = 2), hematologic disorder (*n* = 1), rheumatologic condition (*n* = 1), or other (*n* = 1)

^c^ Upper respiratory tract infection, bronchiolitis, wheezy bronchitis, laryngitis, SARS-CoV-2 infection, or influenza with or without otitis media or other localized bacterial complication

^d^ Fever of unknown origin (*n* = 10), erythema multiforme/allergic reaction (*n* = 3), multisystem inflammatory syndrome in children (MIS-C) (*n* = 1), inflammatory bowel disease (*n* = 1), nonspecific diarrhea of infancy (*n* = 2), periodic fever (*n* = 1)

^e^ Respiratory multiplex polymerase chain reaction (PCR) or antigen detection assay, SARS-CoV-2 PCR assay, combined SARS-CoV-2, respiratory syncytial virus and influenza PCR assay, herpes simplex virus PCR assay, or stool multiplex PCR assay

^f^ Herpes simplex virus or Epstein-Barr virus

^g^ Urine, cerebrospinal fluid, pleural fluid, or abscess fluid

**Supplementary Table 3.** Clinical Characteristics, Diagnoses and Detected Pathogens in 188 ED-Recruited Children According to the Etiologic Group

|  | Bacterial infection  (*n* = 34) | Viral  infection  (*n* = 112) | Viral-bacterial  co-infection  (*n* = 19) | Infection of  undetermined  etiology  (*n* = 15) | Non-infectious disease  (*n* = 8) |
| --- | --- | --- | --- | --- | --- |
| Age (yrs), median (interquartile range, IQR) | 3.0 (0.4–7.4) | 1.5 (0.7–3.0) | 1.9 (1.1–4.3) | 4.1 (1.2–8.6) | 8.5 (2.8–11.3) |
| Chronic conditions, *n* (%) |  |  |  |  |  |
| None | 26 (76.5) | 93 (83.0) | 14 (73.7) | 11 (73.3) | 3 (37.5) |
| Immunosuppressive disease or medication | 3 (8.8) | 2 (1.8) | 1 (5.3) | 1 (6.7) | 0 (0.0) |
| Other condition | 5 (14.7) | 17 (15.2) | 4 (21.1) | 3 (20.0) | 5 (62.5) |
| Disease characteristics, *n* (%) |  |  |  |  |  |
| Antibiotic treatment upon discharge | 28 (82.4) | 6 (5.4) | 18 (94.7) | 7 (46.7) | 0 (0.0) |
| Admitted to hospital | 20 (58.8) | 10 (8.9) | 7 (36.8) | 6 (40.0) | 3 (37.5) |
| Admitted to intensive care unit | 1 (2.9) | 1 (1.9) | 3 (15.8) | 2 (13.3) | 0 (0.0) |
| Clinical diagnoses, *n* (%) |  |  |  |  |  |
| Viral respiratory infection^a^ | 0 (0.0) | 58 (51.8) | 9 (47.4) | 0 (0.0) | 0 (0.0) |
| Undetermined viral infection | 0 (0.0) | 29 (25.9) | 0 (0.0) | 0 (0.0) | 0 (0.0) |
| Pneumonia | 4 (11.8) | 0 (0.0) | 6 (31.6) | 5 (20.0) | 0 (0.0) |
| Pyelonephritis | 14 (41.1) | 0 (0.0) | 2 (10.5) | 0 (0.0) | 0 (0.0) |
| Suspected or microbiologically verified  sepsis without focus | 7 (20.5) | 0 (0.0) | 0 (0.0) | 0 (0.0) | 0 (0.0) |
| Tonsillitis | 0 (0.0) | 10 (9.0) | 1 (5.3) | 1 (6.7) | 0 (0.0) |
| Gastroenteritis | 2 (5.9) | 8 (7.1) | 1 (5.3) | 0 (0.0) | 0 (0.0) |
| Skin or soft tissue infection | 4 (11.8) | 0 (0.0) | 0 (0.0) | 0 (0.0) | 0 (0.0) |
| Osteomyelitis | 1 (2.9) | 0 (0.0) | 0 (0.0) | 0 (0.0) | 0 (0.0) |
| Enteroviral disease, Epstein-Barr virus infection, or  exanthema subitum | 0 (0.0) | 4 (3.6) | 0 (0.0) | 0 (0.0) | 0 (0.0) |
| Central nervous system infection | 1 (2.9) | 1 (0.9) | 0 (0.0) | 1 (6.7) | 0 (0.0) |
| Appendicitis | 1 (2.9) | 0 (0.0) | 0 (0.0) |  | 0 (0.0) |
| Non-infectious disease or fever of unknown origin | 0 (0.0) | 2 (1.8) | 0 (0.0) | 8 (53.3) | 8 (100.0) |
| Any viral sample^b^ collected, *n* (%) | 22 (64.7) | 60 (53.6) | 13 (68.4) | 11 (73.3) | 5 (62.5) |
| Multiplex PCR respiratory sample collected, n (%) | 7 (20.6) | 5 (4.5) | 5 (26.3) | 4 (26.7) | 3 (37.5) |
| Respiratory viruses, *n* |  |  |  |  |  |
| SARS-CoV-2 | 0 | 10 | 4 | 0 | 0 |
| Rhinovirus | 0 | 1 | 3 | 0 | 0 |
| Human bocavirus | 0 | 0 | 0 | 0 | 0 |
| Respiratory syncytial virus A or B | 0 | 1 | 3 | 0 | 0 |
| Parainfluenza virus 1, 2, 3, or 4 | 0 | 1 | 1 | 0 | 0 |
| Human metapneumovirus | 0 | 1 | 1 | 0 | 0 |
| Adenovirus | 0 | 2 | 0 | 0 | 0 |
| Influenza virus A or B | 0 | 1 | 1 | 0 | 0 |
| Coronavirus 229E, NL63, OC43, or HKU1 | 0 | 0 | 0 | 0 | 0 |
| Enterovirus | 0 | 0 | 1 | 0 | 0 |
| Other viruses, *n* |  |  |  |  |  |
| Herpesviruses^c^ | 0 | 1 | 2 | 0 | 0 |
| Astrovirus | 0 | 0 | 1 | 0 | 0 |
| Bacterial species isolated from blood or other sterile site^d^, *n* |  |  |  |  |  |
| *Escherichia coli* | 11 | 0 | 1 | 0 | 0 |
| *Streptococcus* species (*pneumoniae*, *pyogenes*,  *agalactiae*, or *anginosus*) | 1 | 0 | 1 | 0 | 0 |
| *Pseudomonas aeruginosa* | 2 | 0 | 0 | 0 | 0 |
| *Staphylococcus aureus* | 0 | 0 | 0 | 0 | 0 |
| *Enterococcus faecalis* | 1 | 0 | 0 | 0 | 0 |
| *Aerococcus urinae* | 1 | 0 | 0 | 0 | 0 |
| *Enterobacter cloacae complex* | 0 | 0 | 1 | 0 | 0 |
| *Klebsiella pneumoniae complex* | 0 | 0 | 1 | 0 | 0 |
| *Veillonella parvula* | 0 | 0 | 0 | 0 | 0 |
| *Haemophilus parainfluenzae* | 0 | 0 | 0 | 0 | 0 |

^a^ Upper respiratory tract infection, bronchiolitis, wheezy bronchitis, laryngitis, SARS-CoV-2 infection, or influenza with or without otitis media or other localized bacterial complication

^b^ Respiratory multiplex PCR or antigen detection assay, SARS-CoV-2 PCR assay, combined SARS-CoV-2, respiratory syncytial virus and influenza PCR assay, herpes simplex virus PCR assay, or stool multiplex PCR assay

^c^ Herpes simplex virus or Epstein-Barr virus

^d^ Urine, cerebrospinal fluid, pleural fluid, or abscess fluid

**Supplementary Table 4.** Clinical Features of 30 ED-recruited Children with Discordant Results Between the Point-of-Care MxA Result and Adjudicated Etiology of Infection, Based on the Cutoff of 200 µg/L

| POC MxA (µg/L) | Reference MxA (µg/L) | Etiology of Infection | Clinical Diagnosis | Detected Pathogens | Highest CRP (mg/L) |
| --- | --- | --- | --- | --- | --- |
| 112 | 190 | Viral | Tonsillitis | None (negative for *Streptococcus pyogenes*) | 12 |
| < 50 | 86 | Viral | URTI | None | 32 |
| 168 | 190 | Viral | Gastroenteritis / URTI | None | 41 |
| 136 | 147 | Viral | URTI | None | 16 |
| < 50 | 94 | Viral | Undetermined viral infection | None | 24 |
| < 50 | 234 | Viral | Mononucleosis | Epstein-Barr virus | 26 |
| < 50 | 878 | Viral | Undetermined viral infection, suspected roseola | None | 3 |
| 51 | 74 | Viral | URTI | None | 3 |
| 115 | 549 | Viral | Undetermined viral infection | None | 35 |
| 146 | 264 | Viral | Undetermined viral infection | None | 3 |
| 103 | 230 | Viral | Bronchiolitis | None | 8 |
| 64 | 907 | Viral | URTI | None | 14 |
| 181 | 460 | Viral | URTI | None | 13 |
| 75 | 193 | Viral | URTI | None | 61 |
| 127 | 333 | Viral | Obstructive bronchitis | None | 25 |
| 86 | 25 | Viral | Undetermined viral infection | None | 27 |
| 192 | 342 | Viral | URTI | None | 9 |
| 122 | 89 | Viral | Undetermined viral infection | None | 3 |
| 182 | 143 | Viral | Undetermined viral infection | None | 118 |
| 198 | 77 | Viral | URTI | None | 11 |
| 164 | 537 | Viral | Gastroenteritis | None | 3 |
| 170 | 272 | Viral | Undetermined viral infection | None | 3 |
| 118 | 396 | Viral | Gastroenteritis | Adenovirus | 3 |
| 183 | 663 | Co-infection | URTI, otitis media | None | 22 |
| 78 | 254 | Co-infection | Bacterial-type pneumonia | Rhinovirus, parainfluenza virus 3 | 341 |
| 127 | 145 | Co-infection | URTI, perforated otitis media | None | 46 |
| 64 | 113 | Co-infection | URTI, otitis media | None | 52 |
| 25 | 25 | Co-infection | Bacterial-type pneumonia, pleural empyema | *Streptococcus pneumoniae*, SARS-CoV2, rhinovirus | 292 |
| 76 | 59 | Co-infection | Bacterial-type pneumonia | *Pseudomonas aeruginosa*, rhinovirus, enterovirus | 156 |
| 25 | 25 | Co-infection | Bacterial-type pneumonia | SARS-CoV2, influenza A | 126 |

URTI – upper respiratory tract infection

Cases listed in chronological order

**
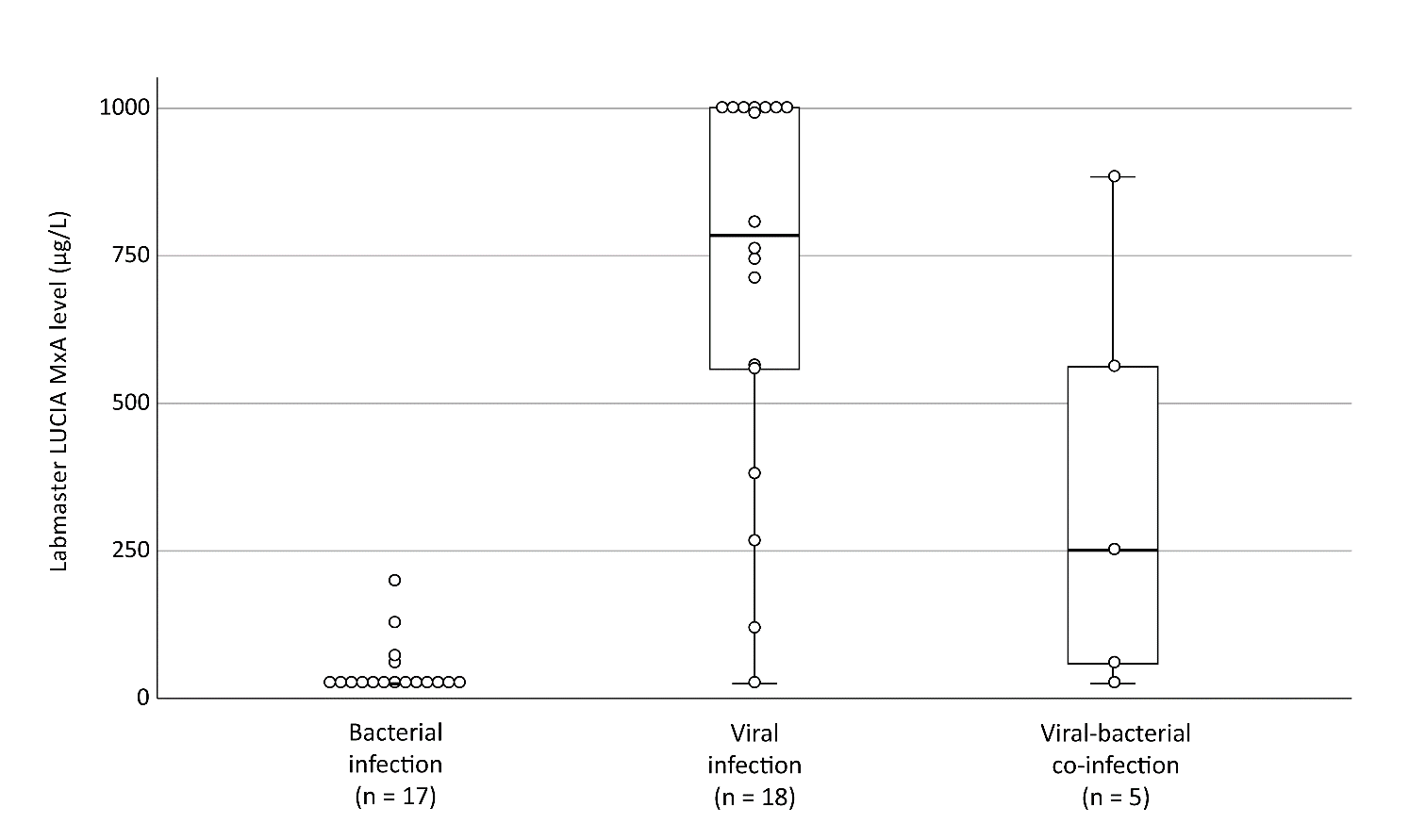
Supplementary Fig. 1** Point-of-Care Labmaster LUCIA MxA Levels in 40 ED-Recruited Children with Microbiologically Confirmed Etiology

For each group, the horizontal line represents the median, the box the upper and lower quartiles, and the whiskers the 95% confidence interval. For pairwise comparisons of groups “Viral infection” and “Bacterial infection”, *P* < 0.001; of groups “Viral infection” and “Viral-bacterial co-infection”, *P* = 0.14; and of groups “Bacterial infection” and “Viral-bacterial co-infection”, *P* = 0.074, respectively, by Mann-Whitney U test.

**
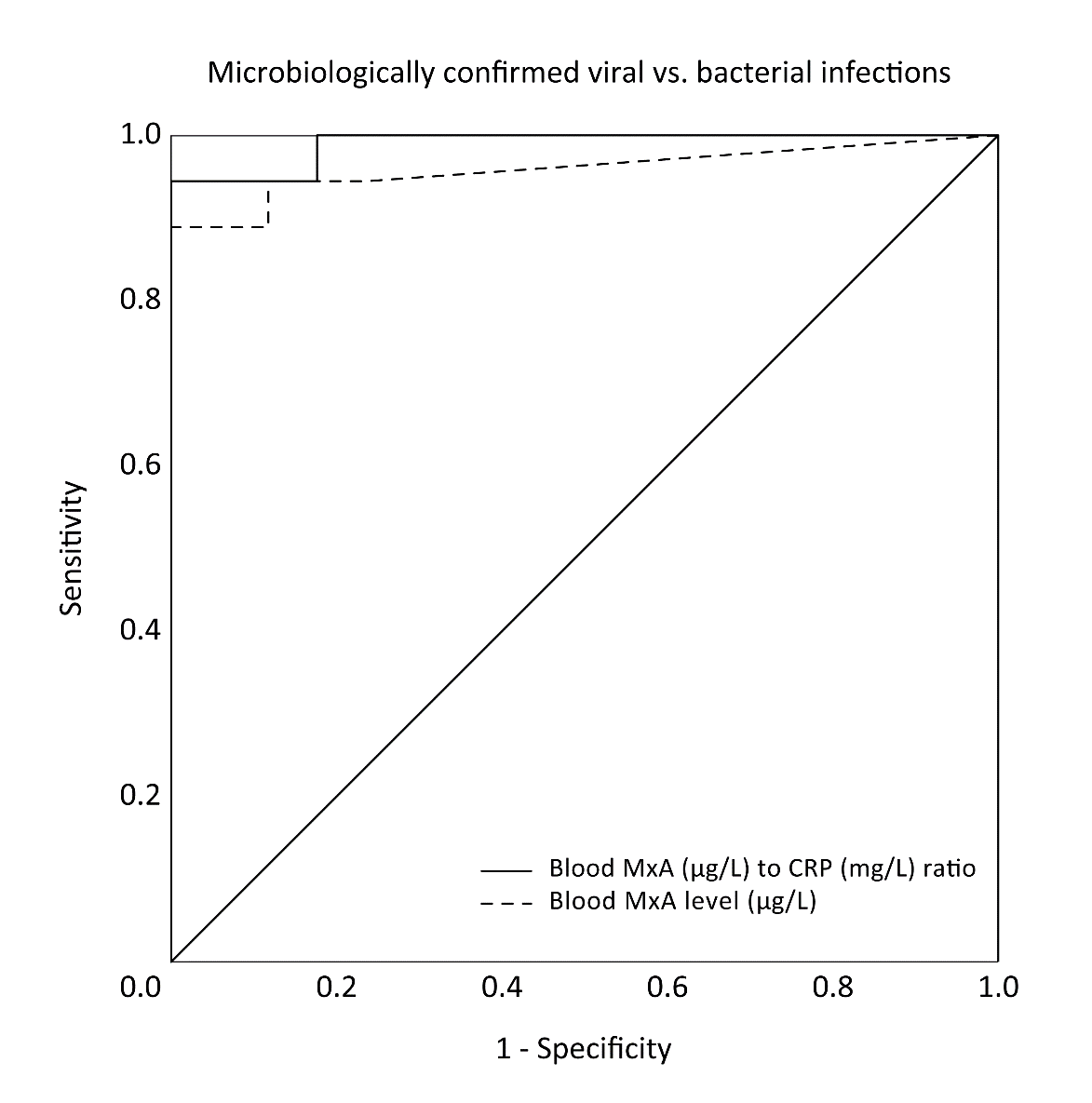
Supplementary Fig. 2** Differentiation between Viral and Bacterial Infections by Point-of-Care Labmaster LUCIA MxA and MxA to CRP Ratio in 40 ED-Recruited Children with Microbiologically Confirmed Etiology

Receiver operating characteristics (ROC) curves for differentiating between microbiologically confirmed viral (n = 18) and bacterial (n = 17) infections in febrile children at the emergency department. Area under the curve for point-of-care blood MxA level and MxA (µg/L) to CRP (mg/L) ratio, 0.96 (95% confidence interval, CI, 0.89–1.00) and 0.99 (95% CI, 0.97–1.00), respectively
